# Vesiculated Long Non-Coding RNAs: Offshore Packages Deciphering Trans-Regulation between Cells, Cancer Progression and Resistance to Therapies

**DOI:** 10.3390/ncrna3010010

**Published:** 2017-02-23

**Authors:** Farah Fatima, Muhammad Nawaz

**Affiliations:** Department of Pathology and Forensic Medicine, Ribeirão Preto Medical School, University of São Paulo, Av. Bandeirantes 3900, Ribeirão Preto 14049-900, Brazil; farah@usp.br

**Keywords:** extracellular vesicles, exRNA, long non-coding RNAs, trans-regulation, RNA interference, cancer senescence, drug resistance, RNA-based therapeutics, biomarkers

## Abstract

Extracellular vesicles (EVs) are nanosized vesicles secreted from virtually all cell types and are thought to transport proteins, lipids and nucleic acids including non-coding RNAs (ncRNAs) between cells. Since, ncRNAs are central to transcriptional regulation during developmental processes; eukaryotes might have evolved novel means of post-transcriptional regulation by trans-locating ncRNAs between cells. EV-mediated transportation of regulatory elements provides a novel source of trans-regulation between cells. In the last decade, studies were mainly focused on microRNAs; however, functions of long ncRNA (lncRNA) have been much less studied. Here, we review the regulatory roles of EV-linked ncRNAs, placing a particular focus on lncRNAs, how they can foster dictated patterns of trans-regulation in recipient cells. This refers to envisaging novel mechanisms of epigenetic regulation, cellular reprogramming and genomic instability elicited in recipient cells, ultimately permitting the generation of cancer initiating cell phenotypes, senescence and resistance to chemotherapies. Conversely, such trans-regulation may introduce RNA interference in recipient cancer cells causing the suppression of oncogenes and anti-apoptotic proteins; thus favoring tumor inhibition. Collectively, understanding these mechanisms could be of great value to EV-based RNA therapeutics achieved through gene manipulation within cancer cells, whereas the ncRNA content of EVs from cancer patients could serve as non-invasive source of diagnostic biomarkers and prognostic indicators in response to therapies.

## 1. Introduction

Extracellular vesicles (EVs) are heterogeneous population of nano-sized vesicles secreted by virtually variety of cell types which based on their size, mode of biogenesis, shape and morphology are categorized into exosomes, microvesicles and apoptotic bodies [1]. Well characterized are exosomes which are produced through the endocytic pathway followed by the fusion of the multivesicular bodies (MVBs) with the plasma membrane and are released into the extracellular environment. This involves several different components of sorting machinery taking place at endosomal compartments and the MVBs (for detailed mechanisms see [1]). The secreted vesicles contain a plethora of bioactive molecules such as nucleic acids including several classes of RNAs [2], proteins and variety of transcriptional factors, lipids, glycans and glycoproteins listed in ExoCarta [3].

Substantial documented evidence has emphasized that EVs transport genetic material between cells and serve as mediators of cell-to-cell communication allowing the exchange of biological information between cells [4,5,6]. Now it is tempting to speculate that EVs in fact mediate bidirectional communication and the transport of regulatory molecules could be observed bidirectionally [7]. EVs carrying diverse cargo can move through biological fluids and thus may elicit long distance inter organ communication by dissemination of their cargo from one place to the other. The secretion and transportation of EVs from biologically active cells may be context dependent relating to certain signals that a particular cell receives such as corresponding to tissue remodeling and regeneration or in response to certain diseases [8]. Due to their natural capacity in transportation and dissemination of abnormal proteins, lipids, mutated genes and deregulated nucleic acids; EVs are implicated in number of diseases.

Non-coding RNAs (ncRNAs) also referred to as non-protein coding RNAs represent part of the genome that does not encode genetic information into proteins. They are broadly categorized into short ncRNAs and long ncRNAs (lncRNAs) or long intergenic ncRNA (lincRNA), encompassing several other ncRNA species such as Piwi-interacting RNAs (piRNAs), short interfering RNAs (siRNAs), small nuclear and nucleolar RNAs (snRNAs, snoRNAs) among others [9,10,11,12]. Approximately 90% of the genome sequence is actively transcribed, but translated proportion is less than 2% of the whole genome [13]. The rest of the proportion has long been considered as a “dark matter”. However, the ENCODE (Encyclopedia of DNA Elements) project has revealed that more than 90% of the human genome contains functional ncRNA [14,15].

The short ncRNA such as microRNAs (miRNAs, miRs) are frequently studied class of ncRNAs and are precisely regulated during developmental processes. It is estimated that approximately 30% of all protein coding genes are regulated by miRNAs and are imperative to shaping the transcriptome of eukaryotic cells [16,17]. The miRNAs are invariably known to regulate gene expression at post transcriptional level governing several cellular pathways related to development [18,19,20,21,22,23,24]. The transcriptional modulation and morphological changes by miRNAs have lately been recognized in governing cellular fates [25].

The lncRNA part of the genome is least well characterized and has complex organization as compared to miRNAs and are thought to be highly regulated in the eukaryotic genome [26]. Gene structure and expression analysis of lncRNA has revealed that more than 9600 human genome loci are classified as lncRNAs; however, less than 100 loci have been characterized for their biological roles in the cell [27,28]. Consequently, it has been believed that ectopic translation and cryptic mRNAs are rare in the human lncRNAome [28]. Describing lncRNAs merely on the basis of lack of protein-coding capability remains far from satisfying, particularly when their heterogeneous functionalities are enormously emerging in recent years.

It is important to emphasize on asking what makes lncRNA functional. Perhaps, the most obvious answer is their structural feature that is characterized by short stem-loop secondary structure; whereas the lncRNA sequences may form complexes with other nucleic acids and their cognate RNA-binding proteins (RBP) inside the cell. The ability of lncRNAs to scaffold into higher degree of organization and tridimensional modular domains could enable protein binding and may permit the recruitment of epigenetic regulators to specific binding targets [26,29]. As such, lncRNAs facilitate the recruitment of histone modifying complexes in a site-specific chromatin contexts [30], by acting in *cis* (at the site of transcription) or *in trans* (at distantly located genes) [31,32]. What is more known about lncRNAs is their ability to regulate transcription indirectly by controlling the subcellular localization of transcription factors. There has been reported several classes of lncRNAs [32]. Given these features, ultimately the functional outcomes of lncRNAs are implicated in chromatin remodeling, splicing, and concomitant development of various diseases including cancer [10,33,34,35,36,37,38,39]. Additionally, such interactions of lncRNAs may promote cellular senescence and dormancy in cancer cells that confer resistance against therapies [40,41,42,43].

Interestingly, recent years have witnessed yet another way of regulatory roles implicated through secretory lncRNAs that are transported to distant locations via EVs. Accumulative data have revealed several classes of lncRNAs detected in EVs (Table 1). Since lncRNAs are able to bind and recruit epigenetic modifiers on specific genomic loci (mentioned above); such roles are also accomplished through EVs that transport and recruit lncRNA machineries and epigenetic modifiers from one cell to the other and may induce epigenetic modifications in recipient cells [44].

## 2. The ncRNA Precursor’s Incorporation into EVs

Over the last decade, different possibilities for ncRNA secretion into extracellular environment have been elucidated. This includes those secreted through EVs or those in association with RBP and high density lipoprotein complexes [45,46,47,48,49,50], as well as passive leakage from cells. However, in pertinent to the presence of extracellular RNA (exRNA) found inside secreted EVs versus outside EVs (i.e., non-EV exRNA) is a debated subject as there are discrepancies in the results shown by different labs [45,50,51,52]. In order to discriminate RNA encapsulated within/or on the surface of EVs from those non EV bound exRNA, it is critical to digest isolated RNA fractions with RNase and proteinase to disrupt ribonucleoproteins and RNA exterior to vesicles [53]. This will deplete non-EV exRNA leaving behind EV-encapsulated RNA.

Not only the ncRNA content in EVs but also the mechanisms by which endogenously expressed RNA species are packaged into EVs is a focus of great interest both in basic research as well as for their prospective therapeutic applications. It is widely established that miRNAs are processed in cytoplasm and readily available for targeting their respective mRNA transcripts or interaction with proteins [54,55,56,57,58]. However, the precise mechanisms of miRNA sorting and packaging into EVs remain poorly understood.

There are initial claims that ribonucleoproteins might have essential role for RNA-sorting into EVs along with few other described factors. Since, RNA-induced silencing complex (RISC) is attributed in directing miRNAs to the target mRNA, the RISC components have recently been proposed in miRNA sorting into EVs. It is tentative that EVs by themselves do not have RISC complex-associated proteins, therefore it could be assumed that only the precursor miRNAs (i.e., pre-miRNAs), but not the mature miRNAs are packaged into EVs have the potential to exhibit biological activity in the recipient cell [59]. However, it is tempted to emerge that the co-localization, and accumulation or re-localization of miRISC components at multivesicular bodies (sites of exosome biogenesis) may favor the processed miRNA sorting into EVs [60,61,62]. A study by Melo et al. further clarified this interim mechanism by emphasizing that tumor cell-derived EVs are loaded with RISC-Loading Complex (RLC) and display cell-independent capacity to process pre-miRNAs into mature miRNAs [63]. The incorporation of pre-miRNAs in association with Dicer, transactivation-responsive RNA-binding protein (TRBP) and Argonaute (AGO2) proteins may potentially drive the processing of pre-miRNAs in a cell-independent manner [63]. Although not fully established, it might be assumed that the presence of RISC components with EV-miRNAs may have more efficient patterns of genetic targeting in recipient cells, since RISC guides miRNAs to target their cognate mRNA in order to govern the regulation of gene expression. However, such EV-sorting mechanisms relating to lncRNAs processing and packaging into EVs are exclusively missing, therefore future studies may warrant better understating of such mechanisms.

## 3. EV-Mediated miRNA Transport and Epigenetic Regulation in Recipient Cells

In 1993, Ambros and colleagues discovered a gene (Lin 4), the gene product of which was a small non-protein coding RNA that negatively regulates the level of LIN-14 protein and affects the developmental process in *Caenorhabditis elegans* [64]. The discovery of gene producing short ncRNA transcripts with no functional open reading frame raised a new debate in transcriptional regulation. As the ncRNAs are expressed endogenously and regulate several cellular processes through regulating gene expression, it could be expected that cell might have evolved yet another novel mechanism of trans-regulation between cells by virtue of transporting ncRNAs via EVs. However, it is critical to understand how EVs enable targeted transport of ncRNAs to responsive cells for targeting the transcriptional machinery and de novo gene regulation within recipient cells.

Initial document indications have revealed that EVs carrying several regulatory factors including ncRNAs travel horizontally to destined target cells which could act within recipient cells and confer epigenetic regulation, cellular reprogramming and the phenotypic modulation of the recipient cells [65,66,67]. More studies keep on demonstrating that EV-mediated mRNA transfer could be translated into proteins within recipient cells and represent novel features of epigenetic gene regulation and cellular reprogramming [68,69,70]. In a similar way, cells could use EVs for shuttling ncRNAs to neighboring cells or distant cells that could find their cognate mRNA sequences in recipient cells. This could manipulate the transcriptome as well as the genetic programs of the recipient cells, implying that the biological effects of recipient cells could be dictated through EV-shuttled ncRNAs.

Sorting of selective patterns of ncRNAs into EVs might serve a purpose for selective functions elicited in recipient cells. For instance, a subset of miRNAs from mesenchymal stem cells (MSCs) is selectively sequestered into EVs which potentially target transcription factors and related genes thereby governing the several cellular pathways such as angiogenesis, cellular transport, apoptosis, and proteolysis in recipient cells [71].

## 4. EV-Mediated Long Non-Coding RNA Transport: A Novel Source of Epigenetic Regulation

In contrast to miRNAs, the mechanisms underlying EV-associated lncRNAs secretion and their biological roles are less described and are only more recently starting to be explored. The lncRNAs could be detected in EV fractions recovered from various sources such as cultured supernatants and body fluids such as blood (plasma, serum) and urine related to different physiological states (Table 1).

Cell free Telomeric Repeat-containing RNA (cfTERRA) lncRNA is detected in EV fractions recovered from mouse normal and tumor tissues, human blood plasma, as well as from supernatants of cultured medium. When incubated with recipient cells, the cfTERRA induced transcription of inflammatory cytokines through telomere dysfunction (genomic instability) of responsive cells. This indicates the extrinsic functions of cfTERRA in the tissue microenvironment [72]. In another parallel study, the elevated levels of cfTERRA were detected in EVs during telomere dysfunction induced by the expression of the dominant negative TRF2 [73]. EVs from these damaged cells were also enriched in DNA damage marker γH2AX and fragmented telomere repeat DNA. It was shown that cfTERRA-containing EVs transport a telomere-associated molecular pattern (TAMP) and telomere-specific *alarmin* from dysfunctional telomeres to the extracellular environment in order to elicit an inflammatory response [73]. Since cfTERRA can be readily detected in human serum it may provide a useful biomarker for the detection of telomere dysfunction in the early stage of cancers and aging-associated inflammatory disease [72,73]. HOX transcript antisense RNA (HOTAIR) lncRNA is also detected in EVs from serum of inflammatory disease such as rheumatoid arthritis (RA) patients, whereby the higher levels are associated with migration of active macrophages [74]. Therefore, the HOTAIR could be a potential biomarker for diagnosing rheumatoid arthritis patients. Likewise, the detection of HOTAIR from body fluids of cancer patients may serve as early tumor diagnosis [75,76]. In addition, the lncRNA profiling in EVs from peripheral blood may offer a novel source of coronary artery disease [77].

It is interesting to consider that the expression pattern of lncRNA molecules are different from their parent cells, which indicate that similar to miRNAs, the lncRNA also represent selective loading into EVs [78,79]. The levels of lncRNAs in EVs best reflect the change of their cellular levels upon exposure of the cells to bleomycin-induced DNA damage [78]. This indicates that in response to genomic instability, cells might exhibit higher expression levels of lncRNAs and manage to secrete highly expressed lncRNAs into EVs. Additionally, the degradation products of lncRNAs from donor cells appear to be enriched in their EVs. Since, the lncRNAs serves as a scaffold for histone modification complexes [29], their delivery to recipient cells might have a role in histone modifications and methylation allowing the epigenetic changes in recipient cell genome [44]. EV-mediated intercellular transport of PARTICLE (triplex forming lncRNA) serves as nuclear genetic platform for transcriptional repression. In fact, PARTICLE forms a DNA-lncRNA triplex upstream to CpG island promoter of methionine adenosyltransferase (*MAT2A*), and facilities *MAT2A* repression via methylation. Such epigenetic interplay of PARTICLE-lncRNA with *MAT2A* is implicated as a recruitment platform for gene-silencing machinery in response to irradiation [44]. It is proposed that a distinct lncRNA profile could be secreted into EVs against certain stimuli, and could be measured from blood or urine [80]. This study implied that Vitamin D signaling (VDS) regulates the expression of certain lncRNAs in a manner consistent with VDS protection against skin cancer, whereas measuring the lncRNA profile from body fluids could serve as skin cancer biomarkers. The lymphoblastoid cells induced with Epstein-Barr virus (EBV) may secrete EVs containing miRNAs and lncRNA such as H19 and H19 antisense, indicating the role of viral RNA transfer via EVs and possible mechanism by which EBV may extend their communications in paracrine fashion [81].

Additionally, the roles of lncRNA transfer may also be implicated in protection against disease as well as in normal physiology of the body. EVs secreted from apoptotic neurons contain high levels of HN12-lncRNA that can be delivered to non-apoptotic cells and can inhibit cell apoptosis in Hirschsprung’s disease (HSCR) by maintaining the function of mitochondria, ATP production and the release of cytochrome C [82]. This indicates the roles of EV-mediated lncRNA transport in intercellular communication for protecting HSCR development. Therefore, the high levels of HN12 in the circulation could serve as a biomarker for early screening of HSCR. Interestingly, the detection of lncRNAs of pseudogenes (presumably, processed pseudogenes) in EVs from saliva of healthy individuals [83], and detection of diverse population of protein-coding gene and lncRNAs in EVs from plasma of a healthy individual [84], could be indicative of expression regulation in normal physiological processes.

## 5. EV-Associated ncRNAs: Conveyers of Genomic Instability and Tumor Progression

For the past two decades, the causes of tumorigenesis have been considered largely as a consequence of genetic and/or epigenetic alterations in protein-coding regions. Taking into account the regulatory feature of ncRNAs, Calin and colleagues for the first time pioneered the idea that miRNAs are involved in human tumorigenesis initially reported in the chronic lymphocytic leukemia [85,86,87]. These pioneering studies clarified that the genomic complexity of the cancer is far greater than expected before.

The last decade has witnessed newly discovered mechanisms of tumor initiation and metastasis facilitated by cell-to-cell communication via EVs [88,89,90,91,92,93]. In fact, EVs could educate certain cells towards tumor initiating phenotypes, and may recruit primary tumor cells to anatomically distinct locations for the construction of premetastatic niche [94]. Notably, EVs act as abettors and facilitators of tumor cells in stromal remodeling and immune evasion [8,95]. It is important to emphasize that metastatic potential of EVs is greatly reliant on EV-mediated transport and dissemination of abnormally expressed regulatory ncRNAs [96,97]. Considerably EV-mediated dissemination of ncRNAs is thought to represent widespread regulatory functions through modulating genetic profiles of recipient cells and may foster genomic instability [72,78,96]. As such, EV-mediated transport of aberrantly expressed ncRNAs may consequently result into construction of premetastatic niche, modulation of tumor microenvironment and cancer progression [96,98,99,100,101,102,103,104,105,106,107].

Astrocyte-derived EVs mediate an intercellular transfer of phosphatase and tensin homolog (PTEN)-targeting miRNAs to primary metastatic tumor cells in order to suppress PTEN, and thus allowing primary tumor cells to become metastatic [96]. This supports the proposition that EVs shuttle miRNAs between tumor cells and their metastatic niche and such reciprocal cross-talk facilitates a mechanism of co-evolution between metastatic cells and their microenvironment during the adaptive metastatic outgrowth. The adoptive metastatic outgrowth could also be observed through metabolic reprograming of tumor microenvironment. The miRNA signatures secreted from breast cancer cells facilitate metastasis by increasing nutrient availability and reprogramming the energy metabolism of non-tumor cells in a given premetastatic niche [99]. Since the nutrition supply to cancer cells is largely dependent on tumor neovasculature, in this context EV-ncRNAs facilitate angiogenesis in a given tumor microenvironment [108,109].

More recently, it has been reported that the miR-7977 in EVs is responsible for hematopoietic dysfunction of MSCs by reducing the levels of poly(rc) binding protein 1. This failure of normal hematopoiesis subsequently permits the progression of myeloid neoplasm [110]. Multiple myeloma (MM)-derived EVs may also transfer oncogenic proteins, and adhesion molecules with reduced level of tumor suppressor miR-15a that favor tumor growth in recipient cells in vitro as well as in vivo [111]. In contrast, cancer cells could deliver miRNAs to stem cells, constituting a reciprocal transfer mechanism. For instance, MM cells deliver miR-146a into MSCs via EVs, which results in an elevated level of cytokine secretion, subsequently facilitating the cell viability and migration of MM cells [112]. This indicates the contribution of EV-miRNA in positive feedback loop between MM cells and MSCs. A similar mode of transfer was observed when lung cancer-derived EVs were co-incubated with MSCs which revealed that EVs from cancer cells could initiate global changes to lncRNA expression in recipient cells [113].

Additionally, the lncRNA with highly conserved sequences known as ultra-conserved lncRNA (ucRNA) are differentially expressed in EVs as compared to their parent hepatocellular carcinoma (HCC) cells, indicating a selective mechanism of ucRNA secretion [114]. The highly expressed ucRNA named TUC339 was detected in EVs from HCCs and was functionally implicated in modulating tumor cell growth. In another example, the CD90^+^ liver cancer cells modulate endothelial cell phenotype and promote angiogenesis through EV-contained H19 lncRNA. However, EVs from parental hepatoma cells lacking CD90 do not exhibit such features, indicating that such features of EV-linked H19 are exclusive to CD90^+^ cancer stem-cell-like cells [115]. More recently the elevated expression level of H19 was observed in cervical cancer cells, and was secreted into cultured supernatant via EVs. H19 lncRNA was shown to promote cell proliferation, multicellular tumor spheroid formation and anchorage-independent growth of cervical cancer cells in vitro [116]. The aberrant expressions of H19 lncRNA are implicated in multiple malignancies where its expression levels correlate with recurrence, metastasis and patient survival. Certain lncRNAs detected from prostate cancer derived EVs are enriched for RBP binding motifs as well as miRNA-seeds preferably for let-7 family members and are implicated in carcinogenesis [117]. Interestingly, enrichment of EV-lncRNAs for miRNA seeds as well as RBP sites suggests their regulatory contribution in cancer development. Importantly, that the detection of lncRNAs in EVs from peripheral blood of cancer patients may allow a minimal invasive tool for tumor diagnosis, such as those measured from cohort of colorectal cancer patients [118].

**Figure 1:**
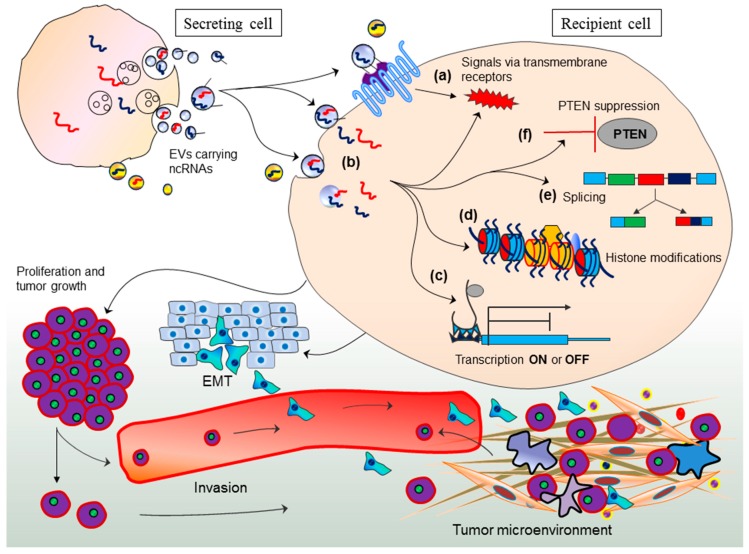
EV-mediated ncRNA transport and mechanism of trans-regulation, genomic instability and tumor progression. Tumor cell-derived EVs carrying ncRNAs are transported to recipient cells and may trigger cellular responses either by (**a**) direct receptor mediated interactions; or (**b**) could be endocytosed, followed by the release of ncRNAs in cytoplasm; (**c**) The ncRNAs may find their target mRNAs in recipient cell cytoplasm and may modulate gene expression either by repressing or activating the target genes; (**d**) The ncRNAs might recruit methylation machinery and may contribute to histone modifications; (**e**) EV-mediated delivery of splicing components may contribute to processing of precursor RNA transcripts in recipient cells and affect the transcription products; (**f**) The ncRNAs may target and inhibit tumor suppressor *PTEN* gene. These means of epigenetic regulation elicited in recipient cells consequently result into genomic instability and global changes in transcriptomic profiles ultimately giving rise to cancer initiating cell phenotypes such as epithelial mesenchymal transition. Collectively, genomic and phenotypic changes may exhibit enhanced proliferation, tumor growth, invasion and modulation of tumor microenvironment. EVs: Extracellular vesicles, ncRNAs: non-coding RNA, EMT: Epithelial mesenchymal transition.

RNA-Seq of RNA content from colorectal cancer cell-derived EVs has revealed numerous RNA species including lncRNAs, miRNAs, splicing/fusion genes, and pseudo gene transcripts differentially distributed in EVs compared to parent cancer cells [119]. Moreover, it is interesting to know the co-existence of U1 and U2 ribonucleoproteins with their cognate snRNAs within EVs [119]. This suggests that EV-mediated transport of splicing cargo may elicit splicing events in recipient cells. Collectively, it could be envisaged that EV-mediated transport of splicing cargo may confer epigenetic regulation in the evolution of diseases and may offer novel therapeutic targets. Evidences suggest that splicing events demonstrate potential linkages with the progression of various diseases including cancer, and may serve as targets of gene therapy [120,121,122,123]. Given all observations together, it is apparent that EV-mediated transport of ncRNAs to recipients cells exhibit several ways to initiate and aggravate cancer (Figure 1).

## 6. EV-ncRNAs Are Extended Messages in Regulating Responses to Chemotherapy

Recent data have shown that aberrant expression of lncRNAs and EV-associated transport serve as newly described mediators of chemotherapeutic response in cancer cells in vitro as well as in vivo. As such EV-linked regulatory RNAs including lincRNA-VLDLR (linc-VLDLR) in human HCC exhibit responses against chemotherapeutic stress, such as sorafenib, camptothecin, and doxorubicin [124]. In fact, the over expressed linc-VLDLR in EVs demonstrate the capacity of lncRNAs to mediate chemotherapeutic stress response in HCC. Two parallel studies, have revealed that long intergenic noncoding RNA, regulator of reprogramming (linc-ROR) is released into EVs from HCC [97,125]. HCC cells facing hypoxic stress may confer high expression levels of linc-RoR that is released into extracellular environment via EVs [97]. This indicates the role of lncRNA secretion or transport via EVs may critical to cope with hypoxia. In another occasion, the TGFβ expression is thought to favor the secretion of linc-RoR RNA via EVs and is associated with chemoresistance in HCC cells. This implies that the effects of TGFβ on chemoresistance in HCC cells involve the linc-RoR-dependent effects on tumor-initiating cells. Therefore, targeting linc-ROR could be a possible strategy to improve chemosensitivity in HCC cells [125]. Additionally, EV-mediated transport of lncRNAs may extend the bystander effects of chemoresistance into chemosensitive cells. As such example was seen where co-incubation of tamoxifen resistant cell-derived EVs with tamoxifen sensitive cells promoted chemoresistance within sensitive cells [126]. This bystander effect is mediated by transfer of EV-linked UCA1 from resistant cells to sensitive cells in vitro.

In addition to lncRNAs, EV-associated miRNAs may also serve as mediators of the chemotherapeutic response. Several examples are documented from breast cancer cells, using EVs as their extended messages for introducing drug resistance or sensitivity in recipient cells by transferring subset of miRNAs [127,128,129,130,131]. MiRNAs from docetaxel-resistant breast cancer cells could be incorporated into EVs, which upon co-incubation with recipient cells induce chemosensitivity, exhibiting bystander effects of resistant cells [128]. Moreover, transport of miR-221/222 via EVs may enhance drug resistance in recipient breast cancer [130].

EV-associated miRNA subsets may also confer therapeutic response against drugs in colon cancer, colorectal cancer and prostate cancer cells in vitro [132,133,134,135]. It has been proposed that loss of miR-200c from 5-fluorouracil resistant colon cancer cells makes lymph endothelial cells more susceptible to invasion in vitro [132]. EV-miRNA levels such as miR-200c and miR-141 are upregulated against decitabine treatment (methylation inhibitor), and could prompt the acquisition of epithelial cell-like characteristics in drug resistant colorectal cancer cells [133]. This indicates that DNA demethylation treatment drives epithelial cell-like characteristics, whereas measuring EV-linked miR-200c and miR-141 levels may serve as indicator candidate for mesenchymal-epithelial transition (MET) in colorectal cancer cells. Recently, the evaluation of EV-associated miRNA expression patterns in hospitalized patients predicted resistance of MM against bortezomib (Bz). Such observation in the patients resistant to Bz is revealed by down-regulated panel of miRNAs such as miR-16-5p, miR-15a-5p, miR-20a-5p, and miR-17-5p [136]. As, the routine workup of MM hardly suggests a value for prediction of drug resistance, the miRNAs content of EVs from patient blood may allow a better understanding of the in vivo environment of MM patients. Likewise chemotherapies, the radiotherapy may also reflect the consequences of EV-mediated miRNA transport during radio-resistance as observed in lung cancer [137,138].

Other examples come from adipocyte-derived EVs. The miRNAs from cancer-associated adipocytes and cancer associated fibroblasts could be transported to ovarian cancer cells via EVs. Such transfer confers the paclitaxel resistance through miRNA targeted suppression of apoptotic peptidase activating factor 1 (APAF1) in recipient cells [139]. EVs from adipose tissue derived MSCs (AD-MSCs) are implicated in promoting chemosensitivity in HCC cells and facilitate antitumor efficacy of chemotherapeutic agents [140]. Upon transfection, the miR-122 is incorporated into AD-MSC secreted EVs and is delivered to HCC cells, resulting in increased sensitivity to chemotherapeutic agents in vitro as well as in vivo [140]. This offers a novel strategy to enhance cancer cell chemosensitivity endorsing the therapeutic potential of stem cell-derived EVs.

An additional layer of chemoresistance is conferred through EV-mediated induction of senescence and dormancy/senescence in cancer cells. For instance, breast cancer cells trigger MSCs to release distinct miRNA contents into EVs, such as miR-222/223 which in turn favor quiescence in a subset of breast cancer cells and confer drug resistance in a positive feedback loop manner [141]. MSC-derived EVs from breast cancer cells contain over expressed miR-23b, which promotes dormancy in metastatic breast cancer cells through the suppression of myristoylated alanine-rich C-kinase substrate (*MARCKS*) gene [142]. EV-mediated transfer of miR-433 and miR-21-3p could promote resistance to paclitaxel and cisplatin respectively; through the induction of cellular senescence in recipient ovarian cancer cells [143,144]. In addition, the expression levels of certain miRNA sets secreted into EVs against various therapies may render prognostic value (Table 2).

Contrary to the natural route of EV-mediated miRNAs implicated in drug responses, the manipulation of RNA content may offer therapeutic benefits. This could be acquired through reactivation of epigenetically silenced miRNAs such as miR-512 and miR-373 which have been shown to induce sensitivity against cisplatin in lung cancer cells and favor tumor inhibition [145]. Induced sensitivity in cells permitted them to exhibit apoptosis, reduced cell proliferation and reduced cell migration in lung cancer cells. Such re-expression of miRNAs may exert cell-autonomous and non-autonomous tumor-suppressive effects in cancer cells indicating benefits of epigenetic cancer therapy.

## 7. Roles in Tumor Inhibition

The role of EVs is not only inferred in tumor progression, however there is the emerging impression that the cargo shipped by EVs could also elicit anti-tumor activities. Recent data show that EVs could deliver apoptotic and anti-proliferative ncRNAs to cancer cells which favor the inhibition of tumor growth. For instance, tumor-suppressive miRNAs secreted by normal cells could be transferred to cancerous cells via EVs and may act as anti-proliferative entities in recipient cancer cells. Of particular note, the induction of growth inhibition through EV-assisted delivery of miR-143 was seen exclusively in cancer cells in vitro and in vivo. This indicates that secretory tumor-suppressive miRNAs could act as a death signal from winners to losers in a cell competitive process [146].

EV-mediated transfer of miR-375 could efficiently block anti-apoptotic protein Bcl-2 in recipient colon cancer cells and facilitates the inhibition of cell proliferation and metastasis [147]. In a similar way, miR-145 from AD-MSC derived EVs could extend the inhibitory effects of MSCs on prostate cancer by blocking the activities Bclxl protein, followed by increased cell apoptosis of prostate cancer cells [148]. Similarly EV-associated miR-29c could induce apoptosis in recipient cancer cells by targeting and down regulating BCL-2 and MCL-1 [149]. It was recently shown that EVs bearing TNF-Related Apoptosis-Inducing Ligand (TRAIL) may transmit pro-apoptotic signals in tumor sites in vivo and induce growth inhibition [150]. However, no significant reduction on tumor mass was observed. Since, the inhibition of Bcl-2 family of anti-apoptotic proteins has been proposed in cancer therapy [151], in this context EVs may serve as vehicles of shipping Bcl-inhibitory miRNAs.

Additionally, there have been proposed other ways of tumor inhibition achieved through suppression of growth factors, and inhibition of angiogenesis and proliferation [152]. EV-mediated delivery of miRNAs that target mRNAs of growth factors, and cell cycle related factors to cancer cells may greatly contribute to tumor inhibition. For instance a set of certain miRNAs is delivered to ovarian cancer cells via EVs and may inhibit the proliferation tendency in vitro [153]. Additionally, EV-mediated delivery of miR-302b in lung cancer have been shown to suppress cancer cell proliferation and migration by targeting TGFβRII [154]. The wide spread role of growth factors in the construction of tumor neovasculature network could benefit targeted therapy through suppression of growth factors central to angiogenesis. Intra-tumor injection of EVs carrying miR-146 significantly reduced glioma xenograft growth in a rat model of primary brain tumor by targeting epidermal growth factor receptor (EGFR) [155]. MSC-derived EVs are enriched in anti-angiogenic miRNAs such as miRNA-16 that is internalized by breast cancer cells and suppress vascular endothelial growth factor (VEGF) expression in recipient tumor cells in vitro as well as in vivo and inhibit angiogenesis [156]. Similarly EV-mediated delivery of selective miRNAs from human liver stem cells (HLSCs) may reprogram HepG2 hepatoma and HCC cells by inhibiting their growth and survival in vitro [157]. Additionally, in vivo intratumor administration of HLSC-derived EVs in a severe combined immune deficiency (SCID) mice model may induce regression of ectopic tumors through the delivery of miRNAs to tumor cells [157]. The antitumor effect of HLSC-derived EVs and miRNA delivery was also observed in tumors other than hepatoma such as lymphoblastoma and glioblastoma, which showed that the delivery of selected miRNAs via EVs efficiently inhibits tumor growth and may stimulate cancer cell apoptosis [157].

This is interesting to consider that both chemosensitive and chemoresistant cancer cells might exhibit ubiquitous release of certain miRNAs via EVs as those shown from ovarian cancer cells [158]. The ubiquitous release of tumor suppressor miR-6126 seems to serve a purpose in tumor suppression through direct targeting of oncogenes in recipient cancer cells [158]. In contrast, the secretion/depletion of tumor suppressor miRNA from donor cells might favor their viability.

Better understanding of EV biogenesis, and means of their dissemination, could benefit the development of methods to inhibit tumor progression. This implies that inhibiting the release of EVs from tumor cells or manipulating the cellular content, EV content and/or EV uptake by recipient cells may favor to minimizing tumor progression. Since, EVs deliver RNA molecules between cells; they can be engineered for siRNA delivery or delivery of anti-tumor miRNAs and even the drugs to cancer cells.

## 8. EV-Based RNA Interference in Targeted Cancer Cells: Vehicle of Gene Therapy

Inspired from their inherent capability in transporting genetic material between cells; EVs have emerged as vehicles of gene delivery. Researchers have loaded exogenous siRNA into EVs administered for various targets and have shown the delivery efficacy of EVs in vitro as well as in vivo [159,160]. However, to best of our knowledge there is no systematic study pertaining to engineering EVs with lncRNAs. Keeping in view the features of EV-mediated transportation of lncRNAs (mentioned in above sections) implicated in genomic instability and transcriptional regulation in recipient cells; EVs might be ideal vectors to silence or activate the targeted genes by delivering exogenous lncRNA.

However, to apply EVs as delivery vectors, first of all an exogenous RNA needs to be loaded into EVs and the loading efficacy must be defined, prior to their internalization into target cells. The additional consideration is the specific targeting, since EVs have surface chemistries compatible with cell receptors, and thus may interact randomly with off-target cells/tissues. Due to these possibilities administered EVs give rise to unpredicted results/effects. Therefore, the uptake and internalization of EVs by proposed target cells remains a critical question. Some of the convincing arguments provided by Hoshino et al., are interesting to consider that EVs could exhibit the organotropism (i.e., to seek target organs) through different forms of surface integrins presented on their surface [90]. However, further studies will warrant the translation of this knowledge for the administration of EV-cargo in organ guided destinations and biodistribution in in vivo models. More refined methods for EV tailoring, as well as routes of administration are prerequisite. In addition, defining the doses to each separate disease type and whether the dose-dependent RNA interference through EV-administered ncRNA in model animal experiments is promising must be determined. Finally, it would be critical to consider whether EV-mediated delivery of RNA molecules to recipient cells foster transient changes or they confer long lasting and permanent effects [7].

## 9. EV-Encapsulated ncRNAs as Diagnostic and Prognostic Biomarkers: At a Glance

There is already evidence for using exRNAs including circulatory miRNAs and lncRNAs (non-vesicle bound exRNA) from human body fluids for determining diagnostic and prognostic roles of RNA signatures related to human diseases [49,161,162,163]. In this context, the presence of ncRNA in EVs may serve an additional platform for biomarker discovery [49]. Proteins and nucleic acids encapsulated within EVs are thought to be more stable against proteases and nucleases that are naturally present in body fluids. The protection of nucleic acids in EVs provides a great advantage of storage conditions as well as handling at adverse physical conditions such as fluctuations in temperature and changes in pH, multiple freeze and thaw cycles, and thus could be appealing source for biomarker development [1]. In particular, the profiling of EV-linked ncRNAs including short ncRNAs such as small nuclear/nucleolar RNAs [164], miRNAs [165,166,167,168,169,170,171,172], as well as lncRNAs from peripheral blood or urine of cancer patients may predict cancer signatures for early diagnosis of specific cancer types [75,76,118,165,173,174,175,176]. Interestingly, the miR-21 one the global tumor marker is found in serum and plasma of various cancer types and may serve as an independent marker of tumor diagnosis [95,177,178,179]. Presumably, the global profiling or selective screening of EV-RNAs against mutations may predict tumor specific signature, whereas the enrichment on ncRNAs within tumor cell-derived EVs could offer a promising platform for developing disease biomarkers.

In addition to their utility in diagnostic platforms, the EV-ncRNAs from serum/plasma may also serve as bona fide signatures of disease prognosis, tumor recurrence and overall survival. This refers to prognostic implications against chemo therapies as well as radio therapies related to several cancer types [44,134,135,138,158,180,181,182,183,184,185,186]. However, it is critical to compare and standardize results of global investigations regarding EV-associated circulating ncRNAs as well as the recommendations for pre-analytic considerations in biomarker discovery.

## 10. Concluding Remarks and Clinical Implications

Since ncRNAs are expressed endogenously and regulate several cellular processes through orchestrating gene expression organization; the secretion of EVs could be envisaged such that the cell might have evolved a mechanism of trans-regulation by shipping ncRNAs via EVs. Taking into account this notion, EVs might implicate to-and-fro delivery of ncRNAs between cells enabling reprograming of cellular genome. This potential could be extended towards tailoring EVs in therapeutic strategies by loading ncRNAs into EVs. Given that the ncRNAs exhibit heterogeneous mechanisms of action, it would be interesting to deeply understand the functional readouts arising from trans-regulatory effects casted at outbox locations (i.e., ncRNA delivered from one cell and acted in other cell). This will help understand the ways by which EV-linked ncRNAs are implicated in trans-regulation and concomitant effects on health and disease. However, the functional role of tumor specific ncRNAs signatures within EVs still needs to be evaluated rigorously.

In addition to understanding the biological readouts, the technical issues related to harvesting EVs, sample source, analytical platforms for characterization and purpose of downstream analysis must also be considered. Several technical hurdles still require an explicit attention. In particular, standard protocols for EV isolation, purification and characterization of different samples is still debatable issue and applications of conventional and high throughput technologies not only represent advantages but also the limitations [1]. The most trivial bottleneck is the lack of standardized methods for collection and processing of bio-fluids for isolation, purification of EV-cargo prerequisite for intended therapeutic applications [53]. The method of choice should be taken into account based on sample type, volume and the yield, integrity, purity of EVs required for specific downstream analysis as the available instrumentation and processing time [1]. This implies whether the sample is derived from cell-culture media or body-fluids, and whether intended for proteomic analysis or genomic profiling.

The low abundance of ncRNAs within circulating EVs as well as the issues regarding integrity of intact RNA species may add additional layer of complexity [53]. Therefore, a development of highly sensitive detection platforms and high-throughput next generation approaches will warrant the implications of EVs in routine biomarker utility as well as therapeutic development with a recently suggested flow sheet to applying for US food and drug administration (FDA) approval [1].

The evaluation of EVs for clinical trials in human diseases is very limited so far [187,188]. Although the number of patients included in these clinical trials is very small, nevertheless there is emerging potential of EVs for their prospective translation from bench to bedside. Pertaining to envisaging EVs from bench to bedside, the Good Manufacturing Practice (i.e., GMP grade EVs) would be highly recommended. Poorly described methods as well as differences in standard operating procedures (SOPs) could make it difficult to compare and standardize the therapeutic effects of EVs attributed in different reports. Such inconsistencies may limit the expectancy of translating EVs into human clinical trials. Moreover, establishing the safety profile of EVs as well as effectiveness in the resolution of complex disorders also needs explicit attention. The heterogeneous nature of a certain disease itself may hinder the consistency to functional readouts of EVs in therapeutic terms such as stochastic nature of metabolic diseases and cancers. There is intensive interest in the field and recent years have witnessed widespread growth in EVs research both in understanding the basic functions of EVs as well as therapeutic implications. Therefore, it is anticipated that the next decade will form the basis of novel targeted therapeutic strategies in EVs arena with significant advances in clinical settings.

## Figures and Tables

**Table 1:** Long non-coding RNAs in extracellular vesicles implicated in epigenetic regulation, tumor progression and drug resistance.

microRNAs	Implications	Reference
TERRA	Genomic instability and transcriptional regulation of inflammatory cytokines	[72,73]
TERRA	DNA damage response and inflammatory responses	[73]
HOTAIR	Inflammatory responses and migration of active macrophages	[74]
HOTAIR	Urinary biomarker for bladder cancer diagnosis and prognosis	[75]
HOTAIR	Serum-based biomarker for laryngeal squamous cell carcinoma	[76]
lincRNA-p21, HOTAIR, ncRNA-CCND1, TUG1, GAS5, MALAT1	Response to DNA damage	[78]
PARTICLE	Methylation, gene silencing and transcriptional repression	[44]
H19 and H19 antisense	Epstein-Barr virus induced expression in immortalized B cells	[81]
HN12 lncRNA	Inhibition of cell apoptosis and maintaining the function of mitochondria in Hirschsprung’s disease	[82]
linc-RoR	Modulation of chemosensitivity in human hepatocellular cancer	[97]
linc-RoR	Modulation of hypoxia-signaling pathways	[125]
UCA1 lncRNA	Enhanced tamoxifen resistance in breast cancer cells	[126]
TUC339	Progression of hepatocellular carcinoma growth	[114]
H19 lncRNA	Modulation of endothelial cell phenotype and tumor angiogenesis	[115]
H19 lncRNA	Proliferation and anchor independent tumor growth of cervical cancer cells	[116]
BCAR4	Serum-based diagnostic and prognostic markers for colorectal cancer	[118]
CRNDE-h	Serum-based biomarker for diagnosis and prognosis of colorectal cancer	[175]

**Table 2:** Potential microRNAs in extracellular vesicles implicated in tumor progression and drug resistance.

microRNAs	Implications	Reference
miR-10b	Breast cancer cell invasion	[98]
miR-122	Glucose metabolism in premetastatic niche and cancer metastasis	[99]
miR-1246	Oral squamous cell carcinoma metastasis	[106]
miR-100-5p, miR-21-5p	Prostate cancer progression and metastasis	[107]
miR-7977	Hematopoietic dysfunction and progression to myeloid neoplasms	[110]
miR-15a	Multiple myeloma progression	[111]
miR-146a	Multiple myeloma cell survival and migration	[112]
miR-221/222	Enhanced drug resistance in breast cancer	[130]
miR-134	Enhanced drug sensitivity and reduction in triple-negative breast cancer aggression	[131]
miR-34a	Response to chemotherapy in prostate cancer, prognostic biomarker	[134]
miR-1290, miR-375	Prognostic markers in castration-resistant prostate cancer	[135]
miR-208a	Radio-resistance in human lung cancer cells	[137]
miR-29a, miR-150	Prognostic markers against lung cancer radiotherapy	[138]
miR-122	Hepatocellular carcinoma chemosensitivity and increased antitumor efficacy of chemotherapeutic agents	[140]
miR-222/223	Dormancy in early stage breast cancer	[141]
miR-23b	Dormancy in metastatic breast cancer cells	[142]
miR-21-3p	Drug resistance in ovarian cancer	[144]
miR-512, miR-373	Sensitivity against drug and lung cancer inhibition	[145]
miR-143	Tumor inhibition	[146]
miR-375	Tumor inhibition	[147]
miR-145	Tumor inhibition	[148]
miR-29c	Tumor inhibition	[149]
miR-16	Suppression of tumor angiogenesis	[156]
miR-451, miR-223, miR-24, miR-125b, miR-31, miR-122	Inhibition of hepatoma growth	[157]
miR-6126	Ovarian cancer metastasis	[158]
miR-1246	Diagnostic and prognostic biomarker for esophageal squamous cell carcinoma	[169]
miR-21	Diagnostic biomarker for esophageal squamous cell carcinoma, human hepatocellular carcinoma, cervical and ovarian cancer	[95,177,178,179]
miR-146b, miR-222	Prognostic marker of recurrence in papillary thyroid cancer	[186]
